# Clinical Presentation and Immunological Characteristics of Plastic Bronchitis in Children With Severe MPP

**DOI:** 10.1111/crj.70232

**Published:** 2026-11-09

**Authors:** Haojie Wang, Lang Yuan, Chao Sun, Zeyu Zeng, Xiaoyan Dong

**Affiliations:** ^1^ Department of Pulmonology, Shanghai Children's Hospital, School of Medicine Shanghai Jiao Tong University Shanghai China

**Keywords:** children, clinical features, plastic bronchitis, risk factors, severe *Mycoplasma pneumoniae* pneumonia

## Abstract

Plastic bronchitis (PB) complicating severe *Mycoplasma pneumoniae* pneumonia (SMPP) increases respiratory failure risk and worsens outcomes. However, clinical and immunological features of PB concurrent with SMPP remain incompletely understood. This study aimed to characterize clinical, immunological, and radiological features of SMPP children with PB and identify independent predictors of PB. A retrospective analysis compared clinical, immune, and imaging data between SMPP children with and without PB. Risk factors were identified and assessed for diagnostic relevance. SMPP children with PB showed prolonged fever duration, higher peak temperature, and more severe respiratory symptoms compared with children with SMPP alone. Immunological findings included decreased peripheral CD4^+^/CD8^+^ ratio, elevated serum IgM, increased bronchoalveolar lavage fluid (BALF) monocyte percentage (M%), and elevated cytokines IL‐6, IL‐8, and IFN‐γ. Univariate and multivariate analyses identified MP resistance, number of affected lung lobes, serum IgM, CD4^+^/CD8^+^ ratio, and BALF M% as independent predictors of PB in SMPP children. Receiver operating characteristic (ROC) analysis demonstrated that fever duration and the number of affected lung lobes showed diagnostic discrimination for PB, with their combination yielding an area under the curve (AUC) of 0.836. The CD4^+^/CD8^+^ ratio, serum IgM level, and BALF M% showed moderate diagnostic discrimination individually, and their combined model yielded an AUC of 0.736. A comprehensive model integrating macrolide resistance, the number of affected lung lobes, CD4^+^/CD8^+^ ratio, serum IgM level, and BALF M% achieved an AUC of 0.868. SMPP children complicated by PB exhibit significant immune dysregulation and extensive pulmonary pathology. Clinical (MP resistance and number of affected lung lobes) and immunological (IgM level, CD4^+^/CD8^+^ ratio, and BALF M%) markers may facilitate early PB diagnosis and timely treatment.

## Graphical abstract

Plastic bronchitis in children with severe *Mycoplasma pneumoniae* pneumonia is associated with immune dysregulation and more extensive pulmonary involvement. Fever duration, extent of lung involvement, serum IgM, CD4^+^/CD8^+^ ratio, and bronchoalveolar lavage fluid monocyte percentage may serve as early predictors of PB in children with severe *Mycoplasma pneumoniae* pneumonia.

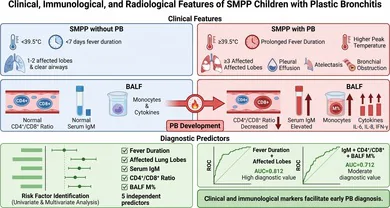

**Abbreviations**
ALP: alkaline phosphatase
ALT: alanine aminotransferase
AST: aspartate aminotransferase
AUC: area under the curve
BALF: bronchoalveolar lavage fluid
CK: creatine kinase
CRP: C‐reactive protein
ESR: erythrocyte sedimentation rate
FDP: fibrin degradation products
GGT: gamma‐glutamyl transferase
LDH: lactate dehydrogenase
M%: monocyte percentage
MP:
*Mycoplasma pneumoniae*

MPP:
*Mycoplasma pneumoniae* pneumonia
N%: neutrophil percentage
PB: plastic bronchitis
PCT: procalcitonin
PLT: platelet count
PT: prothrombin time
ROC: receiver operating characteristic
SMPP: severe MPP
WBC: white blood cell count

## 1 Introduction

*Mycoplasma pneumoniae* pneumonia (MPP) remains one of the leading causes of community‐acquired pneumonia in the pediatric population [1]. Although most MPP cases are self‐limiting, a subset progresses to severe MPP (SMPP), which is defined by persistent high‐grade fever, hypoxemia, radiological worsening, and involvement of extrapulmonary organs [2]. SMPP can even be life‐threatening, particularly in children [3]. Incidence data obtained in northern China showed that a huge number of children are affected by SMPP annually, rising from 0.7% in 2007 to 42.6% in 2016 [4]. In a similar study, incidence of MPP‐associated community‐acquired pneumonia in the United States rose from 2.1 per 1000 pediatric hospitalizations between the years 2018 and 2023 and escalated further up to 12.5 per 1000 by year 2024 [5]. Its increasing prevalence is highly threatening, necessitating early diagnosis and appropriate therapy.

Plastic bronchitis (PB) is a rare condition in which materials produced within the body accumulate in the airways of the lungs, partially or completely obstructing them and leading to reduced airflow and various clinical symptoms [6]. PB can be considered a manifestation associated with congenital heart disease [7], viral respiratory infection [8], and lymphatic disease [9]. However, little is known about the relationship between PB and SMPP. Due to unclear presentations of PB combined with a lack of awareness, timely identification, correct diagnostics, and targeted management are rarely achieved in children with PB, leading to worse outcomes inevitably. A better understanding of PB complicated by MPP, including its clinical features and underlying immune mechanisms, enables physicians to intervene promptly and prevent disease progression.

Earlier studies on the relationship between PB and MPP mainly focused on blood tests and radiologic findings. A large prospective study involving 1169 children with MPP identified 133 PB cases and observed associations with elevated inflammatory markers such as neutrophil percentage (N%), C‐reactive protein (CRP), procalcitonin (PCT), lactate dehydrogenase (LDH), and transaminases, as well as reduced lymphocyte and platelet counts (PLT) [10]. However, immune profiling and airway‐specific inflammatory markers were not assessed [10]. Another retrospective study from Shanghai, which enrolled 296 pediatric children, identified prolonged febrile duration, increased IL‐8 and LDH levels, and decreased complement C3 as independent risk factors for PB [11]. Nonetheless, this study lacked data on bronchoalveolar lavage cytokines, pulmonary MP load, and bronchoscopic features [11], thereby limiting insight into local immune dysregulation. Therefore, a more comprehensive investigation incorporating both systemic and airway‐specific immune responses is warranted to better understand the pathogenesis of PB in children with severe MPP.

To better characterize the clinical features of PB in the context of SMPP, this retrospective study analyzed 231 hospitalized children with confirmed SMPP. Children were grouped based on the presence or absence of PB. Data included clinical symptoms, radiological findings, MPP‐related indicators, routine laboratory tests, coagulation parameters, organ function markers, lymphocyte subpopulations, immunoglobulin levels, complement components, and systemic and alveolar cytokines. By comparing these multidimensional parameters, the study aimed to clarify the distinct clinical and immunological profiles of SMPP concurrent PB and provide evidence to support earlier recognition and more targeted intervention.

## 2 Materials and Methods

### 2.1 Study Design and Participants

This retrospective observational study was conducted at Shanghai Children's Hospital, a tertiary pediatric referral center, and included children diagnosed with SMPP between January 2023 and December 2024. During the study period, 1907 children were diagnosed with MPP, of whom 443 met the diagnostic criteria for SMPP according to the 2023 Chinese National Guidelines for the Diagnosis and Treatment of *Mycoplasma pneumoniae* Pneumonia in Children. Among these 443 children with SMPP, 231 underwent flexible bronchoscopy during hospitalization and constituted the final study cohort. Of these 231 children, 100 were diagnosed with PB and 131 had isolated SMPP without bronchoscopic evidence of PB. The inclusion criteria were as follows: (1) age 0–14 years; (2) diagnosis of SMPP according to the 2023 Chinese national guideline; (3) microbiologically confirmed *M. pneumoniae* infection; (4) complete clinical, laboratory, and immunological data; and (5) availability of flexible bronchoscopic examination for direct assessment of the airways. The exclusion criteria were congenital cardiopulmonary malformations, primary or secondary immunodeficiency, chronic respiratory diseases (e.g., bronchopulmonary dysplasia or cystic fibrosis), and incomplete clinical data. All 231 eligible patients underwent flexible bronchoscopy with bronchoalveolar lavage (BAL) during hospitalization. PB status was determined exclusively according to direct bronchoscopic findings. Patients were classified into the PB group or the isolated SMPP group according to whether a characteristic bronchial cast was directly visualized and removed during bronchoscopy. This study was approved by the Ethics Committee of Shanghai Children's Hospital, Shanghai Jiao Tong University (Approval number: 2025R152‐E01), and conducted in accordance with the Declaration of Helsinki. The requirement for informed consent was waived due to the retrospective nature of the study.

### 2.2 Diagnosis of SMPP

The diagnosis of SMPP was established according to the 2023 Chinese National Guidelines for the Diagnosis and Treatment of *Mycoplasma pneumoniae* Pneumonia in Children. The predefined clinical, laboratory, and radiological criteria included persistent high fever (body temperature of ≥ 39°C for ≥ 5 consecutive days or fever persisting for ≥ 7 days without a downward trend), significant respiratory manifestations, extrapulmonary complications, hypoxemia (SpO_2_ ≤ 93% while breathing room air at rest), characteristic radiological abnormalities, disease progression, and markedly elevated inflammatory or coagulation‐related biomarkers, including CRP, LDH, or D‐dimer. Characteristic radiological abnormalities included homogeneous high‐density consolidation involving ≥ 2/3 of a single pulmonary lobe; high‐density consolidation involving ≥ 2 pulmonary lobes with or without moderate‐to‐large pleural effusion; or diffuse bronchiolitis involving a single lung or ≥ 4/5 of both lungs accompanied by atelectasis caused by mucus plugging. Disease progression was defined as clinical deterioration accompanied by > 50% radiological progression within 24–48 h. *M. pneumoniae* infection was considered microbiologically confirmed when at least one of the following criteria was met: (1) an *M. pneumoniae* IgM antibody titer ≥ 1:160 by the passive agglutination method; (2) a ≥ 4‐fold increase in antibody titer between the acute and convalescent phases; or (3) a positive *M. pneumoniae* DNA PCR result from a nasopharyngeal swab or BAL fluid.

### 2.3 Bronchoscopic Diagnosis of PB

PB was diagnosed based on direct bronchoscopic identification of a bronchial cast. A patient was classified as having PB when bronchoscopy demonstrated an obstructive, cohesive cast within the bronchial lumen that could be removed by bronchoscopic intervention. The cast was characterized by a branching or tubular configuration that reproduced the anatomical architecture of the bronchial tree and had a relatively firm, tenacious consistency, distinguishing it from ordinary airway secretions or mucus plugs. The color of the cast was not considered a diagnostic criterion and could vary from white or yellowish to reddish.

Radiological findings alone, including atelectasis, lobar consolidation, or hyperinflation, were not sufficient to establish the diagnosis of PB. Patients with airway obstruction or atelectasis who underwent bronchoscopy but had no bronchoscopically confirmed bronchial cast were classified as the non‐PB group. Thus, PB was defined by direct bronchoscopic evidence of a characteristic bronchial cast rather than by clinical or radiological findings alone.

### 2.4 Data Collection

Demographic characteristics, clinical manifestations, complications, microbiological findings, imaging findings, laboratory parameters, peripheral immune profiles, serum cytokines, and bronchoalveolar lavage fluid (BALF) findings were retrospectively collected from the medical records. Clinical data included age, sex, duration of fever, peak temperature, hypoxemia, tachypnea, respiratory findings, skin manifestations, and other organ involvement. Microbiological data included *M. pneumoniae* antibody titers, *M. pneumoniae* DNA copy numbers in nasopharyngeal swabs and BALF, and macrolide‐resistance mutation status. Macrolide‐resistance mutations were detected using a multiplex real‐time fluorescence PCR probe assay targeting the *M. pneumoniae* ‐specific gene and the 23S rRNA gene resistance‐associated mutation sites A2063G and A2064G. The assay was performed using a commercial kit from Jiangsu Mole Bioscience Co. Ltd., according to the manufacturer's instructions. Imaging data included chest CT findings, pleural effusion, atelectasis, lung consolidation, number of affected lobes, bronchial obstruction, and bronchiectasis. Laboratory data included complete blood counts, inflammatory markers, liver and myocardial injury markers, and coagulation parameters. Immunological assessments included peripheral T‐, B‐, and NK‐cell subsets, immunoglobulins, complement components, and circulating immune complexes. Serum and BALF cytokine profiles were also collected. The detailed variables and corresponding measurements are presented in the relevant tables.

### 2.5 Univariate and Multivariate Logistic Regression Analyses

PB status was defined as the binary dependent variable. Candidate independent variables were selected from the demographic, clinical, microbiological, imaging, laboratory, immunological, and BALF data collected in this study based on their potential clinical relevance and data availability. Each candidate independent variable was evaluated separately in a univariate logistic regression model to assess its association with PB. The specific independent variables included in the univariate logistic regression analysis are presented in Table 4. Variables with *p* < 0.050.10 in the univariate analysis, or those considered clinically relevant, were subsequently included in the multivariate logistic regression model. A backward stepwise selection procedure was used to identify factors independently associated with PB. Multicollinearity was assessed using variance inflation factors (VIFs), and model fit was evaluated using the Hosmer–Lemeshow goodness‐of‐fit test. The results are presented as odds ratios (ORs) with 95% confidence intervals (CIs) and corresponding *p*‐values.

### 2.6 Statistical Analysis

Patient characteristics were summarized using descriptive statistics. Group comparisons employed Student's *t*‐test or the Mann–Whitney *U* test for continuous variables, and the Yates continuity‐corrected *χ* ^2^ test or Fisher's exact test for categorical variables, as appropriate. Variables with *p* < 0.10 in univariate logistic regression or clinical relevance were included in multivariate logistic regression with backward stepwise selection. Multicollinearity was checked via VIFs, and model fit by the Hosmer–Lemeshow test. For the unified prediction model integrating clinical and immunological variables, a nomogram was constructed to provide an individualized estimate of the probability of PB. ORs with 95% CIs and *p*‐values were reported. Analyses were performed using Python 3.11 (statsmodels, scikit‐learn), and receiver operating characteristic (ROC) curves were generated with R package pROC. Two‐sided *p* < 0.05 indicated significance.

## 3 Results

### 3.1 SMPP Concurrent PB Is Associated With More Severe Clinical Involvement

To investigate the clinical characteristics of SMPP children with concomitant PB, we conducted an analysis comparing the fever characteristics and respiratory manifestations between children with SMPP alone and those with SMPP accompanied by PB. The duration of fever was significantly prolonged in children with SMPP complicated by PB, with a median of 8 days compared with 6 days in those without PB (*p* < 0.001) (Table 1). Moreover, the peak body temperature was higher in SMPP children with PB than in those without PB (Table 1). Compared with non‐PB children, the PB group also showed higher frequencies of hypoxemia (18% vs. 2.3%), tachypnea (19% vs. none), and decreased breath sounds (45.0% vs. 9.2%), all with *p*‐values of < 0.001. Wheezing rales were more common in children with SMPP and PB than in those without PB (*p* = 0.011), and the incidence of wet rales did not differ significantly between the two groups (Table 1). Regarding complications, liver injury, myocardial injury, pericardial effusion, and rash were observed in both groups, but only liver injury (25.0% vs. 6.1%, *p* < 0.001) and myocardial injury (12.0% vs. 3.1%, *p* = 0.017) were significantly higher in children with PB than in those without PB (*p* = 0.014) (Table 1). These findings indicate that SMPP concurrent PB is associated with more severe respiratory manifestations and a higher frequency of complications.

**TABLE 1:** Demographic characteristics, clinical features, and complications in SMPP patients with and without PB.

Characteristics		PB	Non‐PB	*Z*/*χ* ^2^	*p*
Gender, *n* (%)				*χ* ^2^ = 1.65	0.199
Boys		38 (38.0%)	62 (47.3%)		
Girls		62 (62.0%)	69 (52.7%)		
Age, *n* (%)				Fisher	0.173
1 to < 3 years		1 (1.0%)	1 (0.8%)		
3 to < 6 years		22 (22.0%)	37 (28.2%)		
6 to < 9 years		41 (41.0%)	62 (47.3%)		
≥ 9 years		36 (36.0%)	31 (23.7%)		
Decreased breath sounds				*χ* ^2^ = 37.29	**< 0.001**
Yes		45 (45.0%)	12 (9.2%)		
No		55 (55.0%)	119 (90.8%)		
Wet rales				*χ* ^2^ = 0.44	0.508
Yes		48 (48.0%)	56 (42.7%)		
No		52 (52.0%)	75 (57.3%)		
Wheezing rales				Fisher	**0.011**
Yes		9 (9.0%)	2 (1.5%)		
No		91 (91.0%)	129 (98.5%)		
Hypoxemia				*χ* ^2^ = 15.09	**< 0.001**
Yes		18 (18.0%)	3 (2.3%)		
No		82 (82.0%)	128 (97.7%)		
Tachypnea				Fisher	**< 0.001**
Yes		19 (19.0%)	0 (0.0%)		
No		81 (81.0%)	131 (100.0%)		
Duration of fever		8 (7, 10)	6 (5, 7)	*Z* = 8.50	**< 0.001**
Fever peak		39.90 (39.50, 40.00)	39 (38.9–39.5)	*Z* = 7.67	**< 0.001**
Liver injury				*χ* ^2^ = 15.02	**< 0.001**
Yes		25 (25.0%)	8 (6.1%)		
No		75 (75.0%)	123 (93.9%)		
Myocardial injury				*χ* ^2^ = 5.72	**0.017**
Yes		12 (12.0%)	4 (3.1%)		
No		88 (88.0%)	127 (96.9%)		
Pericardial effusion				Fisher	0.169
Yes		4 (4.0%)	1 (0.8%)		
No		96 (96.0%)	130 (99.2%)		
Rash				Fisher	1.000
Yes		4 (4.0%)	5 (3.8%)		
No		96 (96.0%)	126 (96.2%)		
MP pathogen data					
MP antibody titer		1280 (640, 1280)	640 (160, 1280)	*Z* = 6.14	**< 0.001**
NP MP‐DNA copies		825.0 (220.0, 4200.0)	925.0 (56.2, 5760.0)	*Z* = 0.22	0.824
BALF MP‐DNA copies × 10^3^		2295 (497, 9170)	427 (33.93910)	*Z* = 4.13	**< 0.001**
MP resistance				*χ* ^2^ = 45.86	**< 0.001**
Yes		99 (99.0%)	79 (60.3%)		
No		1 (1.0%)	52 (39.7%)		

*Note:* Macrolide resistance was defined as the detection of macrolide resistance‐associated point mutations in respiratory specimens. Categorical variables are presented as *n* (%). For 2 × 2 contingency tables, the Yates continuity‐corrected *χ* ^2^ test was used; Fisher's exact test was used when the theoretical frequency (*T*) was < 5. Continuous variables are presented as median [IQR] and were compared using the Mann–Whitney *U* test. All tests were two‐sided. *p*‐values of < 0.001 are reported as < 0.001.

Abbreviations: BALF, bronchoalveolar lavage fluid; MP, *Mycoplasma pneumoniae* ; NP, nasopharyngeal; PB, plastic bronchitis.

### 3.2 Higher Pathogen Burden and Resistance Are Linked to PB Development

To investigate the association between PB occurrence and pathogen burden as well as MP resistance, we compared serological titers, DNA copy numbers, and resistance rates (Table 1). Children with SMPP and PB exhibited significantly higher MP antibody titers of 1280 compared with 640 in the non‐PB group (*p* < 0.001), and BALF MP‐DNA copy numbers were also elevated, with a median of 2295 × 10^3^ versus 427 × 10^3^ in the non‐PB group (*p* < 0.001) (Table 1). In contrast, nasopharyngeal MP‐DNA levels showed no significant difference between groups, measuring 825 in the PB group and 925 in the non‐PB group (*p* = 0.824) (Table 1). The prevalence of macrolide‐resistant MP was markedly higher in the PB group, reaching 99.0% compared with 60.3% in children without PB (*p* < 0.001) (Table 1). These findings indicate that a high pathogen load in the lower respiratory tract and macrolide resistance are strongly associated with the development of PB in children with SMPP.

### 3.3 SMPP Children With PB Exhibit More Severe and Extensive Pulmonary Abnormalities

A comparative analysis of imaging and bronchoscopy findings was performed to assess differences between SMPP children with and without PB. Pleural effusion was significantly more common in the PB group compared with the non‐PB group (53% vs. 29.8%, *χ* ^2^ = 11.82, *p* < 0.001), as was atelectasis (13% vs. 2.3%, *χ* ^2^ = 8.50, p = 0.004) (Table 2). Lung consolidation was nearly universal in both groups but was more prevalent among children with PB (99% vs. 72.5%, *χ* ^2^ = 27.63, *p* < 0.001) (Table 2). The median number of affected lung lobes was significantly higher in the PB group [3 (IQR 2–4) vs. 2 (1–3), *z* = 6.64, *p* < 0.001]. Notably, bronchial obstruction (14% vs. 0%, *p* < 0.001) was observed exclusively in children with PB (Table 2). No significant difference was found in the incidence of pulmonary embolism between groups (Table 2). These results indicate that SMPP concurrent PB is associated with more severe and extensive pulmonary involvement, which may facilitate earlier diagnosis and guide timely clinical management.

**TABLE 2:** Comparison of clinical, imaging, bronchoscopic, and laboratory characteristics between SMPP patients with and without PB.

Characteristics	PB	Non‐PB	*Z*/*χ* ^2^	*p*
Imaging and bronchoscopy findings				
Pleural effusion			*χ* ^2^ = 11.82	**< 0.001**
Yes	53 (53.0%)	39 (29.8%)		
No	47 (47.0%)	92 (70.2%)		
Atelectasis			*χ* ^2^ = 8.50	**0.004**
Yes	13 (13.0%)	3 (2.3%)		
No	87 (87.0%)	128 (97.7%)		
Lung consolidation			*χ* ^2^ = 27.63	**< 0.001**
Yes	99 (99.0%)	95 (72.5%)		
No	1 (1.0%)	36 (27.5%)		
Number of affected lobe	3.00 (2.00, 4.00)	2.00 (1.00, 3.00)	*Z* = 6.64	**< 0.001**
Pulmonary embolism			Fisher	0.186
Yes	2 (2.0%)	0 (0.0%)		
No	98 (98.0%)	131 (100.0%)		
Bronchial obstruction			Fisher	**< 0.001**
Yes	14 (14.0%)	0 (0.0%)		
No	86 (86.0%)	131 (100.0%)		
Bronchiectasis			Fisher	0.080
Yes	3 (3.0%)	0 (0.0%)		
No	97 (97.0%)	131 (100.0%)		
Hematological and biochemical laboratory findings				
WBC count	7.5 (5.5, 9.9)	7.3 (5.7, 8.8)	*Z* = 0.29	0.771
Neutrophils %	65.8 (55.1, 77.3)	66.3 (56.7, 74.5)	*Z* = 0.03	0.976
Platelet count	311.5 (244.2, 356)	328.5 (268.5, 395.8)	*Z* = −1.15	0.249
CRP	12.5 (5, 28.2)	12 (5, 26)	*Z* = 0.83	0.407
ESR	35 (20, 46)	43 (26, 58)	*Z* = −2.44	**0.015**
PCT	0.1 (0.0, 0.1)	0.1 (0.0, 0.1)	*Z* = 0.17	0.865
Ferritin	136.2 (94.8, 276.9)	105.3 (71, 125.4)	*Z* = 4.12	**< 0.001**
Tissue injury markers				
ALT	22 (13.5, 40)	15 (12, 21)	*Z* = 2.89	**0.004**
AST	35 (26.5, 52.5)	32 (24, 40.5)	*Z* = 2.13	**0.033**
GGT	12.5 (11, 17)	13 (11, 14.5)	*Z* = 0.66	0.511
ALP	125.5 (105.2, 158.8)	154 (130.5, 191.5)	*Z* = −4.50	**< 0.001**
CK	69 (38.5, 135.5)	76 (53.5, 120)	*Z* = −1.12	0.264
CK‐MB	18 (15, 23)	20 (15, 25)	*Z* = −1.21	0.226
LDH	457 (369, 603.5)	332 (282, 374.5)	*Z* = 6.96	**< 0.001**
Coagulation parameters				
Prothrombin time	12.9 (12.2, 13.4)	12.7 (12.1, 13.4)	*Z* = 0.65	0.516
INR	1.1 (1.1, 1.2)	1.1 (1.1, 1.2)	*Z* = 0.62	0.532
APTT	29.3 (27.1, 31.4)	29.6 (27.2, 32.2)	*Z* = −0.77	0.440
Fibrinogen	3.9 (3.0, 4.6)	4.2 (3.7, 4.7)	*Z* = −1.61	0.107
Thrombin time	17.1 (16.4, 17.8)	17.1 (16.4, 17.7)	*Z* = 0.35	0.725
D‐dimer	1.6 (0.8, 4.0)	0.5 (0.3, 0.8)	*Z* = 8.14	**< 0.001**
FDP	5.5 (2.9, 12.3)	2.2 (1.4, 3.5)	*Z* = 6.57	**< 0.001**
Antithrombin III	106.8 (96.8, 112.9)	107.4 (99.8, 118.9)	*Z* = −1.31	0.189

*Note:* Categorical variables are presented as *n* (%). For 2 × 2 contingency tables, the Yates continuity‐corrected *χ* ^2^ test was used; Fisher's exact test was used when the theoretical frequency (*T*) was < 5. Continuous variables are presented as median [IQR] and were compared using the Mann–Whitney *U* test, with *Z* values reported. All tests were two‐sided, and *p* < 0.05 was considered statistically significant. *p*‐values of < 0.001 are reported as < 0.001.

Abbreviations: ALP, alkaline phosphatase; ALT, alanine aminotransferase; APTT, activated partial thromboplastin time; AST, aspartate aminotransferase; CK, creatine kinase; CK‐MB, creatine kinase–MB; CRP, C‐reactive protein; ESR, erythrocyte sedimentation rate; FDP, fibrin degradation products; GGT, gamma‐glutamyl transferase; INR, international normalized ratio; LDH, lactate dehydrogenase; PB, plastic bronchitis; PCT, procalcitonin; SMPP, severe *Mycoplasma pneumoniae* pneumonia; WBC, white blood cell.

### 3.4 SMPP Children With PB Exhibit Elevated Inflammatory Markers and Multiorgan Involvement

To investigate systemic inflammation and tissue damage associated with PB in SMPP children, we compared inflammatory markers, hepatic and myocardial enzyme levels, and coagulation parameters between SMPP children with and without PB. No significant differences were observed in WBC, Neutrophils%, CRP, or PCT levels (*p* > 0.05). However, serum ferritin (*p* < 0.001) and LDH (*p* < 0.001) were significantly elevated in the PB group, indicating heightened systemic inflammation (Table 2). Additionally, alanine aminotransferase (ALT) (*p* = 0.004) and aspartate aminotransferase (AST) (*p* = 0.033) levels were increased, reflecting more pronounced hepatic involvement (Table 2). Markers of coagulation activation, including D‐dimer and fibrin degradation products (FDP), were also significantly higher in children with PB (*p* < 0.001) (Table 2). In contrast, creatine kinase (CK), CK‐MB, gamma‐glutamyl transferase (GGT), and prothrombin time (PT) showed no significant differences between groups (*p* > 0.05) (Table 2). These results indicate that SMPP children complicated by PB exhibit more severe systemic inflammatory responses, hepatic injury, and coagulation activation compared with those without PB, underscoring the increased disease severity.

### 3.5 Decreased Peripheral Lymphocyte Subsets and Elevated Proinflammatory Cytokines in BALF Are Associated With PB in Children With SMPP

To elucidate the immunological characteristics associated with PB in children with SMPP, we compared peripheral lymphocyte subsets, serum immunological markers, and BALF cellular and cytokine profiles between PB and non‐PB groups. In peripheral blood, SMPP children with PB exhibited significantly reduced absolute counts of CD3^+^, CD4^+^, CD8^+^, CD19^+^, and CD16^+^56^+^ lymphocytes (all *p* < 0.05), accompanied by a decreased CD4^+^/CD8^+^ ratio and CD8^+^ (*p* < 0.001), indicating compromised cellular immunity (Table 3). Serum IgA (*p* = 0.001) and IgM (*p* < 0.001) levels were markedly elevated in the PB group, whereas IgG and IgE levels showed no significant differences (*p* > 0.05) (Table 3). CH50 (*p* < 0.001) was significantly decreased, suggesting impaired humoral complement activity.

**TABLE 3:** Peripheral and BALF immune profiles and cytokine levels in SMPP patients with and without PB.

Characteristics	PB	Non‐PB	*Z*	*p*
Blood immune cell subsets				
CD3^+^ (%)	67.4 (59.5, 74.0)	66.2 (59.0, 72.8)	0.54	0.589
CD4^+^ (%)	32.4 (26.4, 38.9)	35.0 (29.8, 39.9)	−2.11	**0.035**
CD8^+^ (%)	28.1 (23.9, 34.9)	25.4 (21.5, 28.5)	3.40	**< 0.001**
CD4^+^/CD8^+^	1.1 (0.8, 1.5)	1.3 (1.2, 1.7)	−3.47	**< 0.001**
CD19^+^ (%)	20.3 (16.1, 26.0)	21.4 (15.4, 26.5)	−0.13	0.899
CD16^+^56^+^/CD3^−^	9.3 (5.9, 13.6)	9.7 (6.6, 13.0)	−0.69	0.491
CD3^+^ (Abs count)	0.9 (0.7, 1.4)	1.3 (0.8, 1.9)	−3.45	**< 0.001**
CD4^+^ (Abs count)	0.5 (0.3, 0.7)	0.7 (0.4, 1.0)	−3.85	**< 0.001**
CD8^+^ (Abs count)	0.4 (0.3, 0.6)	0.5 (0.3, 0.7)	−2.06	**0.040**
CD19^+^ (Abs count)	0.3 (0.2, 0.4)	0.3 (0.2, 0.6)	−2.85	**0.004**
CD16^+^56^+^/CD3^−^ (Abs count)	0.1 (0.1, 0.2)	0.2 (0.1, 0.3)	−4.19	**< 0.001**
Lymphocytes (Abs count)	1.4 (1.1, 1.9)	2.0 (1.3, 2.7)	−3.74	**< 0.001**
Humoral immunity				
Immunoglobulin G (IgG)	10.6 (9.0, 11.9)	10.1 (8.5, 12.3)	1.07	0.282
Immunoglobulin A (IgA)	1.7 (1.3, 2.2)	1.5 (1.0, 1.9)	3.21	**0.001**
Immunoglobulin M (IgM)	1.7 (1.3, 2.4)	1.5 (1.1, 1.7)	3.41	**< 0.001**
Immunoglobulin E (IgE)	111 (39.7, 556.5)	103.5 (38.6, 281)	0.60	0.550
Total complement (CH50)	48.5 (40.8, 53.5)	53.7 (48.5, 61.2)	−4.98	**< 0.001**
Complement C1q	268.1 (216.2, 315.8)	251.3 (206.0, 282.0)	2.22	**0.026**
Complement C4	0.3 (0.2, 0.4)	0.3 (0.2, 0.4)	−0.51	0.610
CIC	5.1 (0.6, 14.8)	3.1 (2.6, 23.9)	−0.20	0.841
Serum cytokines				
IL‐5	4.1 (2.4, 9.2)	2.7 (2.4, 4.3)	4.15	**< 0.001**
IFN‐α	2.8 (2.4, 5.0)	2.7 (2.4, 5.1)	0.86	0.387
IL‐2	2.4 (2.4, 2.9)	2.4 (2.4, 2.8)	2.29	**0.022**
IL‐6	6.1 (3.3, 13.8)	6.3 (3.1, 14.4)	0.01	0.995
IL‐1β	10 (6.5, 15.1)	8.7 (2.7, 25.4)	0.50	0.616
IL‐10	2.4 (2.4, 3.6)	2.4 (2.4, 2.7)	3.03	**0.002**
IFN‐γ	38.3 (14.8, 99.9)	15.5 (7.7, 35.8)	4.35	**< 0.001**
IL‐8	8.9 (2.4, 25.9)	9.9 (2.4, 25.2)	−0.36	0.718
IL‐17	6.4 (3.9, 12.2)	4.1 (2.4, 8.5)	3.49	**< 0.001**
IL‐4	2.4 (2.4, 2.4)	2.4 (2.4, 3.1)	−1.37	0.172
IL‐12P70	2.4 (2.4, 2.6)	2.4 (2.4, 2.8)	0.58	0.564
TNF‐α	6.0 (2.4, 10.2)	5.5 (2.4, 10.0)	0.38	0.703
BALF cytokines & cells				
Nucleated cell count	1573 (785.5, 3218)	1711 (838, 3360.5)	−0.17	0.862
RBC count	2600 (1000, 6600)	1580 (800, 3025)	3.17	**0.002**
N%	63 (52, 71.5)	68 (56.8, 79)	−2.24	**0.025**
L%	13 (10, 18)	13 (9, 20)	−0.17	0.861
M%	20 (15.5, 28.5)	15 (11, 22)	4.31	**< 0.001**
IL‐5	3.2 (2.4, 6.5)	2.7 (2.4, 5.5)	1.37	0.171
IFN‐α	2.4 (2.4, 2.5)	2.4 (2.4, 3.5)	−0.90	0.368
IL‐2	2.9 (2.4, 3.9)	2.4 (2.4, 3.1)	2.63	**0.009**
IL‐6	241.5 (104.4, 595.9)	132.3 (60.4, 354.8)	2.98	**0.003**
IL‐1β	1367.7 (394.9, 3397.4)	733.2 (224.3, 2136.6)	2.41	**0.016**
IL‐10	5.5 (2.6, 12.8)	5.6 (2.4, 18.4)	−0.39	0.695
IFN‐γ	34.8 (14.9, 141.5)	18.4 (5.0, 70.3)	2.60	**0.009**
IL‐8	6377.7 (3577.8, 10 000)	3573.7 (1803.2, 6115.9)	4.50	**< 0.001**
IL‐17	5.3 (3.2, 10.3)	4.5 (2.9, 9.4)	0.85	0.394
IL‐4	2.4 (2.4, 2.7)	2.4 (2.4, 2.5)	1.86	0.063
IL‐12P70	2.4 (2.4, 2.7)	2.4 (2.4, 2.4)	2.18	**0.030**
TNF‐α	45.3 (10.6, 136.4)	15.3 (4.8, 69.5)	2.97	**0.003**

*Note:* Continuous variables are presented as median [IQR] and were compared using the Mann–Whitney *U* test, with *Z* values reported. All tests were two‐sided, and *p* < 0.05 was considered statistically significant. *p*‐values of < 0.001 are reported as < 0.001.

Abbreviations: BALF, bronchoalveolar lavage fluid; CH50, total hemolytic complement; CIC, circulating immune complex; IFN, interferon; IL, interleukin; L%, lymphocyte percentage; M%, monocyte percentage; N%, neutrophil percentage; PB, plastic bronchitis; RBC, red blood cell; SMPP, severe *Mycoplasma pneumoniae* pneumonia; TNF‐α, tumor necrosis factor‐α.

Analysis of BALF revealed more pronounced immunological alterations (Table 3). The monocyte percentage (M%) (*p* < 0.001) and RBC count (*p* = 0.002) were significantly higher in children with PB (Table 3). Among the 12 assessed BALF cytokines, IL‐2, IL‐6, IL‐1β, IL‐8, TNF‐α, IL‐12p70, and IFN‐γ concentrations were significantly elevated in the PB group (all *p* < 0.05), whereas levels of IL‐5, IFN‐α, IL‐17, IL‐10, and IL‐4 did not differ significantly (*p* > 0.05) (Table 3).

These findings suggest that diminished peripheral lymphocyte subsets, increased serum IgM, elevated BALF monocyte proportion, and heightened local proinflammatory cytokine responses—particularly IL‐6, IL‐8, and IFN‐γ—are closely associated with the development of PB in children with SMPP.

### 3.6 Immune and Radiological Variables Independently Predict PB

To identify independent predictors of SMPP concurrent PB, univariate and multivariate logistic regression analyses were performed. Univariate logistic regression identified several variables significantly associated with PB in children with SMPP. Clinically, PB was linked to longer fever duration and higher peak temperatures (Table 4). Radiographic findings such as lung consolidation and multilobar involvement were more common in the PB group (Table 4). Laboratory markers including elevated ferritin, ALT, LDH, D‐dimer, FDP, and reduced ALP and ESR showed strong associations with PB, reflecting systemic inflammation and tissue injury (Table 4). Immune profiling revealed lower counts of CD3^+^, CD4^+^, CD19^+^, and CD16^+^56^+^ lymphocytes, decreased CD4^+^/CD8^+^ ratio, and increased levels of IgA, IgM, IL‐5, IL‐10, IFN‐γ in serum, and IL‐8 and M% in BALF, suggesting both cellular suppression and heightened humoral and cytokine responses (Table 4). Additionally, CRP, PCT, IL‐6, IL‐4, IL‐17, and several BALF cytokines (IL‐2, IL‐6, IFN‐γ, IL‐1β) were not significantly different between groups, indicating selective immune involvement in PB pathogenesis (Table 4).

**TABLE 4:** Univariate logistic regression analysis of risk factors associated with PB in SMPP children.

Character	OR	95% CI	*p*
Decreased breath sounds	8.11	3.98, 16.54	**< 0.001**
Wheezing rales	6.38	1.35, 30.22	0.020
Duration of fever	5.21	3.19, 8.51	**< 0.001**
Fever peak	3.59	2.44, 5.29	**< 0.001**
Liver injury	5.13	2.20, 11.95	**< 0.001**
Myocardial injury	4.33	1.35, 13.86	0.014
Pericardial effusion	5.42	0.60, 49.23	0.134
MP antibody titer	2.45	1.81, 3.31	**< 0.001**
MP resistance	65.16	8.81, 481.84	**< 0.001**
Pleural effusion	2.66	1.55, 4.58	**< 0.001**
Atelectasis	6.38	1.76, 23.04	**0.005**
Lung consolidation	37.52	5.04, 279.11	**< 0.001**
Number of affected lobe	2.87	2.04, 4.05	**< 0.001**
ESR	0.69	0.52, 0.92	**0.011**
PCT	0.81	0.52, 1.28	0.374
CRP	1.10	0.85, 1.43	0.473
Ferritin	1.69	1.08, 2.64	**0.023**
ALT	2.79	1.19, 6.54	**0.018**
AST	3.40	0.91, 12.74	0.069
ALP	0.48	0.33, 0.71	**< 0.001**
LDH	3.86	2.31, 6.45	**< 0.001**
D‐dimer	16.44	5.69, 47.46	**< 0.001**
FDP	9.36	3.52, 24.88	**< 0.001**
CD4^+^ (%)	0.78	0.59, 1.02	0.073
CD8^+^ (%)	1.77	1.32, 2.39	**< 0.001**
CD4^+^/CD8^+^	0.64	0.48, 0.87	**0.004**
CD3^+^ (Abs count)	0.58	0.42, 0.82	**0.002**
CD4^+^ (Abs count)	0.55	0.38, 0.78	**< 0.001**
CD8^+^ (Abs count)	0.78	0.58, 1.04	0.095
CD19^+^ (Abs count)	0.47	0.29, 0.76	**0.002**
CD16^+^56^+^/CD3^−^ (Abs count)	0.63	0.45, 0.88	**0.007**
Lymphocytes (Abs count)	0.53	0.37, 0.76	**< 0.001**
Immunoglobulin A (IgA)	2.15	1.33, 3.46	**0.002**
Immunoglobulin M (IgM)	1.99	1.41, 2.81	**< 0.001**
Total complement (CH50)	0.73	0.42, 1.26	0.257
IL‐5	8.44	2.53, 28.17	**< 0.001**
IL‐10	1.53	1.03, 2.27	**0.035**
IFN‐γ	4.05	1.64, 9.97	**0.002**
IL‐17	1.47	0.92, 2.34	0.105
IL‐4	0.66	0.43, 1.02	0.060
IL‐6	0.90	0.63, 1.29	0.563
BALF N%	0.85	0.65, 1.11	0.230
BALF M%	1.69	1.25, 2.29	**< 0.001**
BALF IL‐5	1.44	0.86, 2.39	0.164
BALF IL‐2	1.15	0.84, 1.59	0.377
BALF IL‐6	1.27	0.90, 1.79	0.169
BALF IL‐1β	1.35	1.00, 1.83	0.052
BALF IFN‐γ	1.33	0.95, 1.86	0.099
BALF IL‐8	2.02	1.47, 2.76	**< 0.001**

*Note:* PB was coded as 1 and non‐PB as 0. For binary variables, odds ratios (ORs) represent the odds of PB for the positive category versus the negative category. Firth penalized logistic regression with Jeffreys prior penalization was used for binary predictors exhibiting complete or quasi‐complete separation. For continuous variables, ORs represent the change in odds associated with a 1‐SD increase in the predictor. ORs are presented with 95% confidence intervals (CIs). All tests were two‐sided, and *p* < 0.05 was considered statistically significant. *p*‐values of < 0.001 are reported as < 0.001.

Abbreviations: PB, plastic bronchitis; SMPP, severe *Mycoplasma pneumoniae* pneumonia.

Multivariable logistic regression analysis was performed using a parsimonious model including MP resistance, the number of affected lung lobes, CD4^+^/CD8^+^ ratio, serum IgM level, and BALF M% (Table 5). These variables remained significantly associated with PB after adjustment for potential confounding factors. To assess the robustness of the findings in view of the strong association between MP resistance and PB observed in the univariate analysis, a sensitivity analysis was performed by additionally forcing MP resistance into the multivariable model. In this five‐variable model, MP resistance showed a strong association with PB (OR = 145.58; 95% CI, 8.53–2484.98; *p* < 0.001). Importantly, the associations of the number of affected lung lobes (OR = 2.087; 95% CI, 1.418–3.074; *p* < 0.001), CD4^+^/CD8^+^ ratio (OR = 0.473; 95% CI, 0.229–0.974; *p* = 0.042), serum IgM level (OR = 2.398; 95% CI, 1.424–4.039; *p* = 0.001), and BALF M% (OR = 1.081; 95% CI, 1.032–1.133; *p* = 0.001) remained statistically significant. These findings indicate that the associations between the main clinical and immunological variables and PB were robust to the inclusion of MP resistance in the multivariable model.

**TABLE 5:** Multivariate logistic regression analysis of independent predictors for PB in SMPP children.

Character	OR	95% CI	*p*
MP resistance	145.58	8.53, 2484.98	**< 0.001**
Number of affected lobe	2.087	1.418, 3.074	**< 0.001**
CD4^+^/CD8^+^	0.473	0.229, 0.974	**0.042**
Immunoglobulin M (IgM)	2.398	1.424, 4.039	**0.001**
BALF M%	1.081	1.032, 1.133	**0.001**

*Note:* ORs represent adjusted odds ratios from the multivariable logistic regression model, with 95% confidence intervals (CIs). For continuous variables, ORs represent the change in odds associated with a 1‐SD increase in the predictor. PB was coded as 1 and non‐PB as 0. All tests were two‐sided, and *p* < 0.05 was considered statistically significant. *p*‐values of < 0.001 are reported as < 0.001.

Abbreviations: BALF, bronchoalveolar lavage fluid; CI, confidence interval; M%, monocyte percentage; OR, odds ratio; PB, plastic bronchitis; SMPP, severe *Mycoplasma pneumoniae* pneumonia.

These findings indicate that, in SMPP children with PB, prolonged fever, extensive pulmonary involvement, elevated serum IgM, reduced peripheral CD4^+^/CD8^+^ ratio increased BALF monocyte proportion, and MP resistance are associated with the presence of PB.

### 3.7 Combined ROC Analysis Highlights Diagnostic Value of Clinical and Immune Markers for SMPP Concurrent PB

To further assess the diagnostic value of key predictors identified through multivariate logistic regression, ROC curve analysis was performed. Among clinical parameters, fever duration demonstrated an area under the curve (AUC) of 0.808, with a specificity of 0.866 and sensitivity of 0.618, whereas the number of affected lung lobes yielded an AUC of 0.728, specificity of 0.714, and sensitivity of 0.652 (Figure 1A). The combination of these two variables improved the AUC to 0.836, with specificity of 0.732 and sensitivity of 0.809, indicating enhanced overall discriminatory ability compared with either parameter alone (Figure 1B).

**FIGURE 1:**
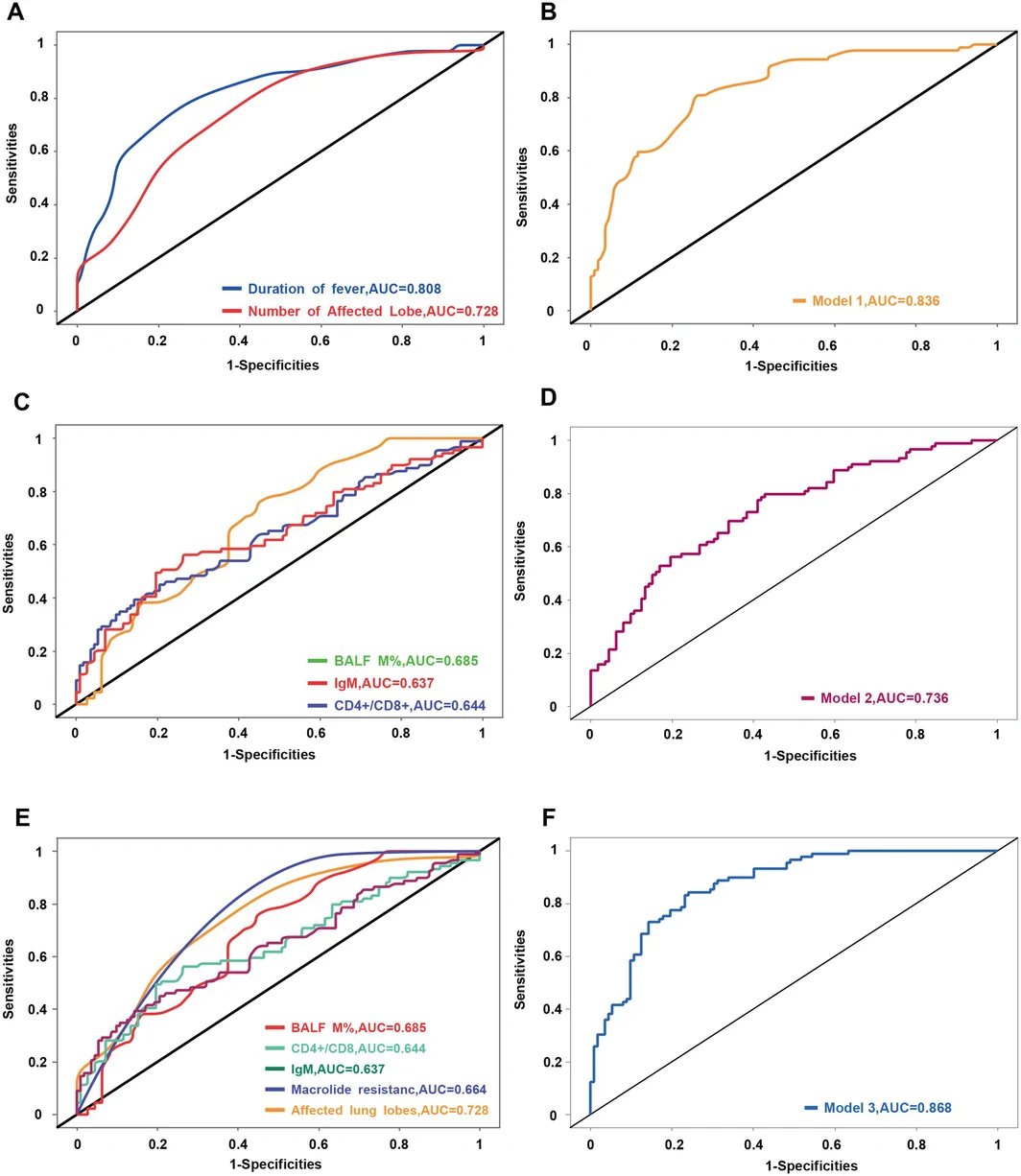
Receiver operating characteristic (ROC) curves for predicting plastic bronchitis (PB) in children with severe *Mycoplasma pneumoniae* pneumonia (SMPP). (A) ROC curves of two single clinical predictors: Duration of fever and number of affected lobes. (B) ROC curve of Combined Model 1 (integrating the duration of fever and number of affected lobes). (C) ROC curves of three single laboratory predictors: BALF M%, serum IgM, and CD4^+^/CD8^+^ ratio. (D) ROC curve of Combined Model 2 (integrating BALF M%, serum IgM, and CD4^+^/CD8^+^ ratio). (E) ROC curves of five single predictors: macrolide resistance, BALF M%, serum IgM, CD4^+^/CD8^+^ ratio, and affected lobes. (F) ROC curve of the multivariable model integrating macrolide resistance mutation, the number of affected pulmonary lobes, the CD4^+^/CD8^+^ ratio, BALF M%, and serum IgM level.

Regarding immune‐related markers, the CD4^+^/CD8^+^ ratio (AUC = 0.644), serum IgM level (AUC = 0.637), and BALF M% (AUC = 0.685) exhibited moderate diagnostic performance (Figure 1C). Their specificities were 0.732, 0.563, and 0.589, and sensitivities were 0.562, 0.618, and 0.697, respectively (Figure 1C). Integration of these three immune parameters into a composite model yielded an AUC of 0.736, with specificity of 0.661 and sensitivity of 0.697 (Figure 1D).

We next evaluated the diagnostic performance of the individual predictors included in the comprehensive model. In addition to the clinical and immune parameters, macrolide resistance mutation was assessed as an individual predictor. Macrolide resistance mutation showed a relatively high diagnostic performance, with an AUC of 0.664, a specificity of 0.339, and a sensitivity of 0.988 (Figure 1E). A multivariable ROC model integrating macrolide resistance mutation, the number of affected lung lobes, the CD4^+^/CD8^+^ ratio, BALF M%, and serum IgM level was constructed to assess their combined diagnostic value. The combined model achieved an AUC of 0.868, with a specificity of 0.768 and sensitivity of 0.832 (Figure 1F).

To further determine clinically useful thresholds, the Youden index was calculated for each candidate predictor (Table S1). The optimal cut‐off for fever duration was > 8 days, yielding a sensitivity of 64.0%, specificity of 87.8%, and Youden index of 0.518. Other optimal cut‐offs included ≥ 3 affected pulmonary lobes (sensitivity, 66.7%; specificity, 72.5%), CD4^+^/CD8^+^ < 1.18 (sensitivity, 56.7%; specificity, 72.2%), M% > 16% (sensitivity, 74.7%; specificity, 53.0%), and serum IgM > 1.92 (sensitivity, 40.0%; specificity, 84.1%). For macrolide resistance, the optimal Youden point yielded a sensitivity of 99.0% and specificity of 39.2%. Among the individual predictors, fever duration of > 8 days had the highest Youden index, suggesting that it may serve as a clinically useful warning threshold for PB.

The calibration performance of the three logistic regression models was further evaluated using calibration curves (Figure S1). The Hosmer–Lemeshow goodness‐of‐fit test showed no significant deviation between the predicted and observed probabilities for Model 1 (*p* = 0.809), Model 2 (*p* = 0.442), or Model 3 (*p* = 0.874), indicating acceptable calibration across all three models. A nomogram based on the multivariable model was subsequently constructed to provide an individualized estimation of the probability of SMPP concurrent with PB (Figure S2).

Overall, prolonged fever duration and greater pulmonary involvement showed the strongest discriminatory performance among the clinical parameters, whereas the CD4^+^/CD8^+^ ratio, serum IgM level, and BALF M% provided additional discriminatory value when considered jointly. These findings suggest that integrating clinical, microbiological, and immune characteristics may aid in identifying PB among children with SMPP.

## 4 Discussion

PB represents a clinically challenging and potentially life‐threatening complication in pediatric children with SMPP [12]. Despite growing awareness of SMPP and its associated complications, early recognition of PB remains limited in routine pediatric practice [13]. In our study, we retrospectively analyzed 131 cases of simple SMPP and 100 cases of SMPP complicated by PB. We found that children with SMPP and concurrent PB showed more severe respiratory manifestations, more extensive radiologic involvement, immune dysregulation, and a pronounced airway inflammatory response. These findings provide a theoretical foundation for the diagnosis of PB in children with SMPP.

Previous investigations have shown that PB in *M. pneumoniae* infection is frequently associated with pronounced clinical manifestations and radiologic abnormalities, implying a strong correlation with disease severity [14]. In line with earlier findings, our cohort demonstrated that children who developed PB experienced longer febrile periods and higher peak temperatures, reflecting a sustained systemic inflammatory response. Respiratory distress was also more evident, with higher rates of hypoxemia, tachypnea, and reduced breath sounds, suggesting significant lower airway obstruction. Radiologically, consolidation, pleural effusion, atelectasis, and bronchial blockage were more common in PB cases, often involving multiple lobes. Such findings are consistent with the mechanical effects of bronchial casts, which compromise ventilation and amplify inflammation within the lung parenchyma. Taken together, these data indicate that PB in SMPP is linked to extensive pulmonary damage and heightened systemic inflammation, clearly distinguishing it from uncomplicated cases.

Immune dysregulation has been recognized as a central mechanism in the pathogenesis of SMPP and its complications, including PB, with both cellular and humoral components implicated [15, 16]. Our study further validated this hypothesis; children with PB showed significantly lower absolute counts of CD3^+^, CD4^+^, CD8^+^, CD19^+^, and CD16^+^56^+^ lymphocytes, a decreased CD4^+^ proportion, an increased CD8^+^ proportion, and a markedly decreased CD4^+^/CD8^+^ ratio. A previous study indicated that a decreased CD4^+^/CD8^+^ ratio is a predictive factor for PB in MPP children, but it only included 39 MPP children with PB [17]. The current study expanded the sample size to 100 PB children, further validating this conclusion. Meanwhile, we found that children with SMPP complicated by PB exhibited significant elevations in serum immunoglobulins, including IgM and IgA. Notably, serum IgM showed potential as a diagnostic biomarker for PB. This is the first study to explore the diagnostic value of elevated IgM in SMPP children with PB. Collectively, these observations provide novel insights into the mechanisms underlying PB initiation and may help guide the development of new therapeutic strategies.

Compared with indicators from peripheral blood, BALF reflects local airway status more directly [18]. In our analysis, children with PB exhibited a higher proportion of monocytes and elevated levels of proinflammatory cytokines, including IL‐2, IL‐6, IL‐1β, IL‐8, and IFN‐γ, compared with those without PB. These cytokines promote epithelial cell damage and excessive mucus secretion, facilitating PB formation [19]. Previous studies have shown that a nomogram incorporating clinical features and BALF IL‐6 levels can be used for PB risk assessment and management in MPP following initial bronchoscopy [20]. Interestingly, the proportion of monocytes in BALF was also identified as an independent predictor of PB development in SMPP children in this research. These findings highlight the diagnostic value of BALF examination in identifying high‐risk SMPP children for PB in clinical practice.

Despite the strengths of this study, several limitations should be acknowledged. First, this was a single‐center retrospective study with potential selection bias. During the study period, 1907 children were hospitalized with MPP, including 443 with SMPP, but only those who underwent bronchoscopy were included. Therefore, the relatively high proportion of PB in our cohort should not be interpreted as the true incidence of PB among all children with SMPP, and the findings may not be generalizable to less severe patients who did not undergo bronchoscopy. Second, BALF M% was measured from samples obtained during bronchoscopy, at which time PB was simultaneously confirmed. Thus, BALF M% should be interpreted cautiously as a marker of local airway inflammation in confirmed PB rather than a feasible pre‐bronchoscopy predictor. Third, the retrospective design precluded assessment of temporal changes in immune responses, and mechanistic studies were not performed; therefore, causal relationships cannot be established. Fourth, the highly imbalanced distribution of macrolide‐resistance mutations resulted in an unstable effect estimate with a wide CI. We have therefore revised the interpretation of this finding to avoid overstatement. Finally, treatment‐related confounding could not be fully controlled. Prospective multicenter studies with consecutive SMPP patients, longitudinal immune assessment, and appropriately timed biomarkers are warranted to validate these findings.

In summary, the concept of PB complicating SMPP encompasses characteristics such as prolonged fever duration, disrupted immune homeostasis, and exacerbated airway inflammation. Incorporating clinical parameters, such as fever duration and the extent of radiologic lung disease, together with immunologic parameters like the CD4^+^/CD8^+^ ratio, serum IgM levels, and BALF monocytosis, could further enhance the early identification of children who may progress to PB. Early diagnosis of these children, based on these features, will provide an earlier opportunity for bronchoscopy assessment and specific immunomodulatory treatment for PB, thereby improving their overall outcomes in the setting of SMPP complicated by PB.

## Author Contributions

**Haojie Wang:** conceptualization, methodology, performed experiments, writing – original draft preparation. **Lang Yuan:** performed experiments, analyzed data, interpreted results of experiments. **Chao Sun:** performed experiments, validation, data analysis, software. **Zeyu Zeng:** software, data analysis. **Xiaoyan Dong:** conceptualization, writing – reviewing and editing, supervision.

## Funding

The authors have nothing to report.

## Ethics Statement

Commencement of this study was predicated upon the receipt of Ethics Committee of Shanghai Children's Hospital, Shanghai Jiao Tong University (Approval number: 2025R152‐E01).

## Consent

The requirement for informed consent was waived due to the retrospective nature of the study.

## Conflicts of Interest

The authors declare no conflicts of interest.

## Supporting information


**Figure S1:** Calibration curves of the three logistic regression models for predicting SMPP concurrent with PB. (A) Calibration curve of Model 1, incorporating fever duration and the number of affected pulmonary lobes. (B) Calibration curve of Model 2, incorporating the CD4^+^/CD8^+^ ratio, BALF M%, and serum IgM level. (C) Calibration curve of Model 3, incorporating macrolide resistance mutation, the number of affected pulmonary lobes, the CD4^+^/CD8^+^ ratio, BALF M%, and serum IgM level. The calibration curves show the agreement between predicted and observed probabilities of SMPP concurrent with PB. The Hosmer–Lemeshow goodness‐of‐fit test yielded *p* = 0.809, 0.442, and 0.874 for Models 1, 2, and 3, respectively.

**Figure S2:** Nomogram for predicting the probability of SMPP concurrent with PB. The nomogram was constructed based on Model 3, incorporating macrolide resistance mutation, the number of affected pulmonary lobes, the CD4^+^/CD8^+^ ratio, BALF M%, and serum IgM level. Each predictor is assigned a corresponding score, and the total score can be used to estimate the individualized probability of SMPP concurrent with PB.

**Table S1:** Optimal cut‐off values of clinical predictors for PB.

## Data Availability

The data that support the findings of this study are available from the corresponding author upon reasonable request.
