# Supplementary material for: Clinical Presentation and Immunological Characteristics of Plastic Bronchitis in Children With Severe MPP
Source: Clin Respir J. 2026 Nov 9;20(11):e70232. doi: 10.1111/crj.70232 (PMC13747718; doi:10.1111/crj.70232)
Supplement: Supplementary file 1 [file CRJ-20-e70232-s003.docx]

Table S1 Optimal cut-off values of clinical predictors for PB

| **Predictor** | **AUC** | **Optimal cut-off** | **Sensitivity** | **Specificity** | **Youden index** |
| --- | --- | --- | --- | --- | --- |
| Fever duration | 0.822 | >8 days | 64.0% | 87.8% | 0.518 |
| Number of affected lobes | 0.745 | ≥3 | 66.7% | 72.5% | 0.392 |
| MP resistance | 0.691 | Resistant | 99.0% | 39.2% | 0.382 |
| CD4^+^/CD8^+^ | 0.638 | <1.18 | 56.7% | 72.2% | 0.289 |
| BALF M% | 0.670 | >16% | 74.7% | 53.0% | 0.277 |
| IgM | 0.636 | >1.92 | 40.0% | 84.1% | 0.241 |
